# Suicide Mortality Patterns in Greek Work Force before and during the Economic Crisis

**DOI:** 10.3390/ijerph16030469

**Published:** 2019-02-06

**Authors:** Evangelos C. Alexopoulos, Katerina Kavalidou, Fani Messolora

**Affiliations:** 1 Occupational Health Department, Metropolitan General Hospital, 15562 Athens, Greece; 2 Suicidal Behaviour Research Lab, Institute of Health and Wellbeing, University of Glasgow, Glasgow G12 OXH, UK; k.kavalidou@gmail.com; 3 Peristeri’s Regional Health Unit, Social Insurance Institute (IKA), 12131 Athens, Greece; messolora@gmail.com

**Keywords:** suicide, comparative mortality ratio, occupation, employment, psychosocial stressors, unemployment, insecurity

## Abstract

*Background*: The global recession of 2007 has attracted research attention in regard to a possible increase of deaths by suicide among employed populations. The aim of the current study was to update the first Greek study on suicide mortality among broad occupational groups during 2000–2009, with the last available data covering the first period of economic crisis and recession in Greece. *Methods*: Data on suicide deaths for the age groups of 15–39, 40–49 and 50–59, between 2000–2013 were retrieved from the national statististical authority of Greece, ELSTAT. The coding of suicide used was X60–X84 (intentional self-harm), based on the 10th International Classification of Diseases (ICD-10). Comparative mortality ratio (CMR) and exact 95% confidence intervals (CI) are presented. *Results*: Males and females in the occupational group of clerks exhibited high and increased CMRs during the crisis period (2010–2013). Although high ratios for males in elementary, agricultural and fishery and armed forces occupational groups were monitored during the whole period, a decrease was evident during the crisis period. Increased trends in CMRs during the crisis were monitored for both males and females in the broad occupational group of members including managers, executives and directors. In addition, females especially in the 50–59 age group showed increased ratios and trends in several occupational groups during the crisis, especially in technologists and associate professionals, plant and machine operators and assemblers, professionals, and craft and related trade workers. *Conclusions*: Austerity-related stress should alert key stakeholders and provide mental health and suicide prevention interventions for employed occupations.

## 1. Introduction

The need of workplace suicide prevention policies has been highlighted through evidence-based studies on the association of different occupational categories with a risk of suicidal behavior and suicide [1,2,3]. Death by suicide for farmers, military and medical personnel has been associated with the easy access to lethal means of suicide, noting that work-specific access to means may influence the choice of a suicide method [4,5,6,7,8]. A retrospective mortality study in Australia presented an association of work-related access to means with the risk of suicide, with women presenting a greater risk compared to men [5].

A number of studies have presented a variety of occupation-related contributory factors in the risk of suicidal behaviors and suicide, such as work-related stressors, lack of full-time employment, occupational trajectories and job insecurity [3,9,10,11]. The most recent comprehensive review on suicide-related outcomes among employed populations, has indicated that occupations with a high proportion of male employees and low-skilled categories have the highest risk of suicide, likely, among other reasons, due to work-related psychosocial stressors [3].

Considering that the global recession of 2007 has attracted research attention in regard to a possible increase of deaths by suicide among unemployed and employed populations [12], there is an emerging need of epidemiological data on the relationship of occupational categories and suicide. Based on the economic crisis and recession period of Greece, the aim of the current study was to update the first Greek study on suicide mortality among broad occupational groups during 2000–2009 [13], with the last available data of 2010–2013.

## 2. Materials and Methods

For the present study we followed the same methodology as in our previous study on the suicide mortality ratios of Greek occupational groups between 2000–2009 [13].

The coding of occupational categories was based on the 2008 version of the International Standard Classification of Occupations (ISCO-88 [14]) for both genders and included: (0) armed forces (unclassified persons); (1) managers, executives, and directors (members); (2) professionals; (3) technologists and associate professionals; (4) clerks; (5) service and market sales workers; (6) skilled agricultural and fishery workers; (7) craft and related trades workers; (8) plant and machine operator assemblers; and (9) elementary occupations (Table 1). Based on the labor force survey conducted by the statistical authority of Greece (ELSTAT) [15], demographic and occupational data were collected. The male and female population distribution by occupational group are presented in the Supplementary Files as seen in Tables S1 and S2.

The number of deaths by suicide for the years 2000–2013 was retrieved from ELSTAT, which includes all the death certificates given by the forensic medicine and criminal investigation authorities of the country. The coding of suicide used was X60–X84 (intentional self-harm) and is based on the 10th International Classification of Diseases ICD-10, [16]. In regard to the calculation of suicide rates by age and occupation group, national statistics were used for the denominator. We conducted our analysis for the working-age groups: 15–39 years, 40–49 years, and 50–59 years. The ratio of standardized cumulative incidences of each group (i.e., the ratio of the number of deaths per year, sex, age group per occupational category divided by the relevant population of occupational group) to the total incidence (i.e., irrespective of occupational group) was defined as the comparative mortality ratio (CMR). The exact 95% confidence intervals (CI) were calculated assuming a Poisson distribution of the observed number of cases. The statistical analysis was performed with SPSS version 24 (IBM Corp., Armonk, NY, USA).

## 3. Results

Males and females in the occupational group of clerks exhibited the higher and increased CMRs in the crisis period (2010–2013) as seen in Table 2 and Table S3. High total CMRs for males were further found for elementary, agricultural and fishery and armed forces occupational groups, but a decrease was evident during the crisis period (2010–2013) compared with the pre-crisis one (2000–2009) shown in Table 2 and Table S3. Increased trends in CMRs during the crisis were monitored for both males and females in the occupational group of managers, executives and directors (members) as seen in Table S3. In addition, females showed increased trends in CMR during the crisis in several occupational groups, especially in armed forces, technologists and associate professionals, skilled agricultural and fishery workers, and plant and machine operators and assemblers (Table S3).

Although any age pattern was not so clear in males, the 50–59 group in females showed higher CMRs in several occupational groups during the crisis (Table 3, Table 4, Table S4a,b, and Table S5a,b).

Higher CMRs were found for all the working-age groups of male members (managers, executives, and directors) in 2012 (Table 3) and those aged 50 to 59 years within the armed forces group in 2010 (Table S4b). Males in elementary occupations show higher CMRs in the age group of 50–59 years in 2012 and 2013 (Table S4b). Increased CMRs were found for males clerks in all age groups, in the whole crisis period with a slight decline in 2011 (Table 3).

In the male members group, higher CMRs were monitored mainly in the younger age group (15–39 years) in the pre-crisis period, while under crisis (2011–2012) the older groups were also affected (Table 3 and Table S4a,b). Male clerks in the age group of 50–59 years were mostly affected between 2000–2009. During the crisis all age groups of clerks were affected (Table 3 and Table S4a,b). In contrary with the 2000–2009 period, in which the highest CMRs in male armed forces were in the age group of 50–59 years after 2010 was not the case (Table S4a,b).

In 2010 high CMRs were monitored for the female plant and machine operators and assemblers, in the age groups of 15–39 and 50–59 years (Table 4). High CMRs (athough did not reach stistically significant levels in all cases) were also evident in professionals in 2010 (CMR: 5.41, 95% CI: 0.91–17.86). 

High CMRs were also found for female technologists and associate professionals and skilled agricultural and fishery workers in 2011 in the age group of 50–59 years; for craft and related trade workers in 2012 in the age group of 50–59 years; and for the armed forces female group in 2013 in the age group of 15–39 years (Table 4 and Table S5b).

In the female members group, higher CMRs were monitored mainly in the younger age group (15–39 years) in the pre-crisis period, while under crisis (2011–2013) members of the younger age group had decreased ratios but older groups were affected (Table 4 and Table S5a,b). In the female clerks group, in which the age group of 50–59 years was mostly affected between 2000–2009, during the crisis the 40–49 group was mostly affected (Table 4 and Table S5a,b). Steadily increased CMRs were found for the oldest age group of female professionals, as seen in Table S5a,b.

Among male occupational groups, significantly low (protective) ratios of suicide mortality during crisis were observed for professionals, technologists and associate professionals, service workers and market sale workers and plant and machine operators and assemblers. Among female occupational groups, protective CMRs were observed for service and market sale workers and elementary occupations (Table S3, Table S4a,b, and Table S5a,b).

## 4. Discussion

The current study presents the suicide mortality ratios of employed men and women in Greece, before and during the global financial crisis, which started to affect Greece in late 2009. The pattern of suicide mortality has considerably changed over the duration of the crisis. While males in armed forces, agriculture and elementary occupations had an elevated risk of suicide overall, a significant decline in their ratios was evident between 2010 and 2013. Results are consistent with employment and social inequality literature. Working-age females were affected in various occupational groups, especially in the age group of 50–59 years old. This latter finding pinpoints the vulnerability of females close to retirement.

The occupational groups of male and female clerks had increased suicide mortality during crisis. Increased trends in CMRs during the crisis were also monitored for both male and female managers, executives and directors (occupational group of members). In both male and female clerks, the age group of 50–59 years was mostly affected during 2000–2009. During the crisis, all age groups of clerks were affected in males, while the 40–49 age group was mostly affected in females. Male members (managers, executives, and directors) exhibited higher ratios in all age groups, while before the crisis higher CMRs were mainly monitored in the younger age group. Concerning female members, the older group was mainly affected during the crisis, while the younger one had decreased ratios.

When examining high and moderate skilled-level categories, research indicates that managers and clerks have a lower risk of suicide, especially when compared to agricultural and elementary groups [3]. Although Australian findings have presented a low risk of suicide for managerial groups, during the post-2008 recessional period, a South Korean study indicated that managers (members) and senior officials had an elevated risk of suicide, compared to the period before the recession (prerecession period: RR = 0.75, 95% CI 0.57–0.99; during recession: RR = 2.81, 95% CI 2.22–3.55) [17]. In consistence with this latter study, all the male age-groups in the category of managers in Greece had increased CMRs during the austerity period of 2012. Furthermore, during the recession, Greek elementary workers had a decline in their ratios. As Milner and LaMontagne recently concluded (2018), suicide risk can vary depending on the country and time of an investigation. As a result, no firm conclusions on the occupational suicide risk differences between regions can be given [3].

The broad clerical occupational group has not been highlighted as a high-risk group for suicide systematically, however when focusing on the pre-crisis and recession period, clerks in South Korea have been presented with the biggest increase in suicide deaths after 2009, compared to other occupations [17,18]. Our study indicated an increased suicide risk for clerks, and our findings overall underscore this group’s constant risk within the employed populations of Greece. Given the elevated risk of suicide among moderate skilled-level populations, possible explanations for the risk in clerks could be job insecurity, which is related to the employee’s underused specialization skills or the high demand and low-payment imbalance [3,19]. Furthermore, this group may possibly include a higher proportion of people with severe chronic health conditions, while it is likely that clerks and office workers may have a misclassification in death certificates, compared to other occupational groups [20,21,22]. As the current study cannot suggest any causal association, in-depth studies should further examine the association of suicide risk and the job-related stressors among clerical workers in Greece.

Those working in armed forces and agriculture have an elevated risk of suicide, partly, due to the access to lethal means. In the same time, the masculinity norms of the army and farm-related occupations seem to be associated with low levels of help-seeking and substance self-medication [4]. In the current study, males in armed forces and agricultural workers had an increased risk of suicide until 2009; however, a significant decrease was found for specific categories in the years 2010–2013. Although there are few studies conducted on the occupational suicide risk during the recession years, our results on the agricultural risk are consistent with a Japanese study that presents a significant suicide risk decline for farmers after 2008 [9,23]. Considering the lack of studies on the risk of suicide among the agricultural employed populations of financially weak countries, cross-cultural studies should investigate the occupational suicide risk of weak versus stronger economies, during recessional periods.

In sharp contrast with males, females showed increased trends in suicide mortality ratios during the crisis in most occupational groups, especially in armed forces, technologists and associate professionals, skilled agricultural and fishery workers, and plant and machine operators and assemblers. Perhaps under financial crisis, the role of socio-cultural adaptiveness based on parental investment theory puts more load in females compared to men [24].

Due to the low female population in several occupational groups such as armed forces, craft and related trade workers and plant and machine operators and assemblers, it was difficult for CMR to reach a statistically significant level, given the low number of events. It should be noted, however, that very high CMRs were monitored in elder females (50–59 age group) in plant and machine operators and assemblers (2010), in professionals (2010), in technologists and associate professionals and skilled agricultural and fishery workers (2011), in the craft and related trade workers (2012) and steadily in clerks and members. High ratios were also monitored in young females (15–39 years) in armed forces (2013) and in plant and machine operators and assemblers (2010). In terms of the gender occupational risk of suicide, Greek women between the ages of 50 to 59 seem to have an increase in their risk during the austerity years of 2010 and 2012. Considering the need of research on the occupational risk of suicide between sexes, further studies should investigate whether the extent of the recent economic crisis differentiated the risk of suicide between employed females and males [25].

Finally, it is worth mentioning that protective ratios of suicide mortality were observed for male professionals, technologists and associate professionals, service workers and market sale workers and plant and machine operators and assemblers. Among female occupational groups, protective ratios were observed for service and market sale workers and elementary occupations.

### Strengths and Limitations

Current findings provide further evidence on the occupational suicide risk during the Greek recession years after 2009. Analyzing national coronial reports of definite suicides for a period of 14 years is the major strength of the current paper. Given the lack of studies on the risk of suicide among occupational groups based on weak economies, the current study sheds light on the possible effects of the recession. In addition, this study assesses different skill-level groups and their vulnerability to suicide in a fragile economical period.

Nevertheless, our findings should be viewed through its limitations. The observational nature of this study cannot provide evidence on the causal relationship of recession and suicide ratios’ trends. Although not likely, we cannot rule out that coronial records may have suffered a limited misclassification bias, especially for specific occupational groups that may have been falsely used in death certificates. In addition, it is possible that in some cases, data on occupation were missing, and so they were not classified under the occupational categories. In order to minimize this effect, we excluded those older than 60 years [13] from the age groups. In any case, the possible lack of classification would be anticipated to be non-differential among the different occupational categories. Differential underreporting is also highly unlikely although time discrepancies from corresponding data caused by delays of other agencies may occur [13]. 

## 5. Conclusions

The gender and age patterns of suicide mortality have considerably changed in the duration of the crisis. Increased trends of suicide mortality ratios during the crisis were monitored for male and female clerks and members (managers, executives and directors). Decreased trends were evident for males in the armed forces, agriculture, and elementary occupations while protective ratios of suicide mortality were observed for male professionals, technologists and associate professionals, service workers and market sale workers, and plant and machine operators and assemblers. 

Females showed increased trends in suicide mortality ratios during the crisis in most occupational groups, especially in armed forces, technologists and associate professionals, skilled agricultural and fishery workers, and plant and machine operators and assemblers while protective ratios were observed for female service and market sale workers and elementary occupations.

All age groups were affected for male clerks and members during the crisis while in most female occupational groups, the 50–59 age group was mainly affected. Overall, the current study highlighted that working-age females of older age were mostly affected during the crisis. Even though Greece is one of a few European countries without a national suicide prevention policy, the austerity-related stress should alert key stakeholders and provide mental health and suicide interventions among employed occupations. Further studies should focus on the effect of prolonged recessional measures on the risk of suicidal behaviors and suicide among the employed population of Greece.

## Figures and Tables

**Table 1:** Occupational categories based on the International Standard Classification of Occupations (ISCO; version 2008).

2-LEVEL	Category of Occupation	Subclasses of Occupation
1 (1)	Unclassified persons	Armed forces, other unclassified persons
3 (3)	Managers, executives, directors (members)	Employers, directors, managers and executives of public administration, big and small public and private enterprises and organisms
7 (4)	Professionals	Scientists (mathematicians, physicians, biologists, architects, engineers, lawyers, teachers and relevant professions) and persons that practise scientific, artistic and relevant professions
4 (4)	Technologists and associate professionals	Technologists and technicians of sciences (natural, health, engineering, etc.) and relevant professions. Specialists on sales, stock broking, agent, services and relevant profession.
2 (2)	Clerks	Clerks, employees of service of customers
3 (2)	Service workers and market sale workers	Occupied in the benefit of personal services. Occupied in the benefit of benefit of services of protection. Models, salesmen and practicing in relevant professions.
7 (1)	Skilled agricultural and fishery workers	Specialized farmers (any type of culture), specialized cattle breeders, bird-breeders, foresters, wood-cutters, fishermen and relevant professions
8 (4)	Craft and related trade workers	Miners, construction workers, metal workers, welders, mechanics, workers in food industry, craftsmen in typo, wood, furniture, textile, clothing, and relevant profession
8 (3)	Plant and machine operators and assemblers	Machine or equipment operators and assemblers in industry (metal, chemical, wood, printing, textile, food, drink, smoke, etc.) and relevant professions, professional drivers
3 (3)	Elementary occupations	Unskilled workers in agriculture, fishing, etc. Unskilled workers in mines, manufacture, transportation, etc. Peddlers, etc.

**Table 2:** Total comparative mortality ratios (CMRs) and 95% confidence intervals due to suicide by occupational group in males and females aged 15–59 years during 2000–2013.

Occupational Categories	Males	Females
Armed forces (unclassified persons)	1.56 (1.20–1.99) *	0.96 (0.05–4.73)
Managers, executives, directors	0.96 (0.84–1.09)	1.29 (0.84–1.91)
Professionals	0.49 (0.41–0.57)	1.04 (0.79–1.32)
Technologists and associate professionals	0.45 (0.35–0.57)	0.63 (0.39–0.97)
Clerks	**2.23 (2.01–2.46)**	**1.33 (1.03–1.68)**
Service workers and market sale workers	0.58 (0.49–0.67)	0.26 (0.15–0.41)
Skilled agricultural and fishery workers	**1.77 (1.61–1.95)**	1.09 (0.79–1.48)
Craft and related trade workers	0.65 (0.58–0.73)	0.48 (0.17–1.06)
Plant and machine operators and assemblers	0.53 (0.44–0.63)	0.60 (0.15–1.63)
Elementary occupations	**2.08 (1.82–2.37)**	0.49 (0.28–0.80)

* Data are presented as CMR (95% CI); CI, Confidence Interval; CMR, Comparative Mortality Ratio; in bold statistically significant results.

**Table 3:** CMRs due to suicide in males during 2010–2013 per occupational category (selected groups based on the pre-crisis CMRs are presented).

Occupational Category	Age Groups	2010	2011	2012	2013
Members (managers, executives, and directors)	15–39	1.31 (0.53–2.73)	1.38 (0.35–3.75)	**2.77 (1.02–6.14)**	0.89 (0.15–2.93)
	40–49	0.53 (0.17–1.29)	1.65 (0.72–3.27)	**2.35 (1.31–3.92)**	1.30 (0.52–2.70)
	50–59	0.84 (0.37–1.66)	1.83 (0.85–3.47)	**2.16 (1.00–4.10)**	0.40 (0.07–1.34)
Clerks	15–39	**3.81 (2.26–6.06)**	1.64 (0.76–3.12)	**2.58 (1.36–4.48)**	2.08 (0.97–3.96)
	40–49	**2.43 (1.23–4.33)**	1.89 (0.92–3.47)	**3.63 (2.28–5.51)**	**2.12 (1.11–3.68)**
	50–59	2.04 (0.99–3.74)	**2.33 (1.29–3.88)**	**2.31 (1.13–4.24)**	**2.21 (1.08–4.05)**
Skilled agricultural and fishery workers	15–39	1.69 (0.74–3.33)	1.03 (0.38–2.29)	1.50 (0.65–2.96)	1.46 (0.64–2.88)
	40–49	1.78 (0.90–3.17)	**2.14 (1.19–3.56)**	1.12 (0.54–2.05)	1.17 (0.57–2.15)
	50–59	1.71 (0.95–2.85)	**1.91 (1.15–2.99)**	1.73 (0.96–2.88)	**1.83 (1.10–2.87)**

Data are presented as CMR (95% CI); CI, Confidence Interval; CMR, Comparative Mortality Ratio; in bold statistically significant results.

**Table 4:** CMRs due to suicide in females during 2010–2013 per occupational category (selected groups based on the pre-crisis CMRs are presented).

Occupational Category	Age Groups	2010	2011	2012	2013
Members (managers, executives, and directors)	15–39	3.04 (0.15–14.99)	*	*	*
	40–49	*	4.53 (0.23–22.32)	*	2.20 (0.11–10.86)
	50–59	*	8.62 (0.43–42.52)	4.54 (0.23–22.42)	*
Technologists and associate professionals	50–59	*	**8.66 (1.45–28.6)**	2.60 (0.13–12.84)	*
Clerks	40–49	2.81 (0.47–9.28)	2.43 (0.62–6.62)	2.93 (0.74–7.97)	*
	50–59	*	*	3.23 (0.54–10.66)	*
Skilled agricultural and fishery workers	40–49	2.00 (0.10–9.84)	*	1.21 (0.06–5.98)	*
	50–59	*	**4.77 (1.51–11.5)**	0.81 (0.04–3.98)	2.14 (0.68–5.15)
Plant and machine operators and assemblers	15–39	12.66 (0.63–62.43)	*	*	*
	50–59	**28.57 (1.43–140.9)**	*	*	*

Data are presented as CMR (95% CI); CI, Confidence Interval; CMR, Comparative Mortality Ratio; in bold statistically significant results * No deaths by suicide registered to the Greek Statistical Authority (ELSTAT).

## Supplementary Materials

The following are available online at http://www.mdpi.com/1660-4601/16/3/469/s1, Table S1: Male population (in thousands) by occupational group during the study period (2000–2013), Table S2: Female population (in thousands) by occupational group during the study period (2000–2013), Table S3: Total CMRs (and 95% CI) due to suicide by occupational group in males and females aged 15–59 years during the pre-crisis (2000–2009 *) and the total study period (2000–2013), Table S4a: Comparative mortality ratios (and 95% confidence intervals) due to suicide by occupational group in males during 2000–2009, Table S4b: Comparative mortality ratios (and 95% confidence intervals) due to suicide by occupational group in males during 2000–2009, Table S5a: Comparative mortality ratios (and 95% confidence intervals) due to suicide by occupational group in females during 2000–2009, Table S5b. Comparative mortality ratios (and 95% confidence intervals) due to suicide by occupational group in females during 2000–2009.
