# Supplementary material for: Suicide Mortality Patterns in Greek Work Force before and during the Economic Crisis
Source: Int J Environ Res Public Health. 2019 Feb 6;16(3):469. doi: 10.3390/ijerph16030469 (PMC6388265; doi:10.3390/ijerph16030469)
Supplement: Supplementary file 1 [file ijerph-16-00469-s001.docx]

**Table S1.** Male population (in thousands) by occupational group during the study period (2000–2013)

| **Category of profession** | **2000** | **2001** | **2002** | **2003** | **2004** | **2005** | **2006** | **2007** | **2008** | **2009** | **2010** | **2011** | **2012** | **2013** |
| --- | --- | --- | --- | --- | --- | --- | --- | --- | --- | --- | --- | --- | --- | --- |
| **Armed forces (unclassified persons)** | 37.3 | 38.0 | 36.5 | 37.1 | 54.7 | 54.4 | 49.2 | 53.3 | 50.6 | 50.5 | 55,2 | 57,1 | 50,0 | 52,7 |
| **Managers, executives, directors (members)** | 303.8 | 289.4 | 310.7 | 294.7 | 335.5 | 332.3 | 336.4 | 327.3 | 343.0 | 326.9 | 307,2 | 127,8 | 118,7 | 127,0 |
| **Professionals** | 257.3 | 267.6 | 274.2 | 276.2 | 316.1 | 317.5 | 331.8 | 334.2 | 343.6 | 334.8 | 327,4 | 348,2 | 341,1 | 334,9 |
| **Technologists & associate professionals** | 143.6 | 141.0 | 159.5 | 163.8 | 172.1 | 175.4 | 189.9 | 200.3 | 196.0 | 190.8 | 193,1 | 162,6 | 145,8 | 136,9 |
| **Clerks** | 197.8 | 199.4 | 189.7 | 191.2 | 202.3 | 209.9 | 208.1 | 200.2 | 210.3 | 195.1 | 190,8 | 182,1 | 160,2 | 147,5 |
| **Service workers & market sale workers** | 260.7 | 265.3 | 268.9 | 291.9 | 276.7 | 284.6 | 227.2 | 286.4 | 290.6 | 297.5 | 289,3 | 445,2 | 404,8 | 383,3 |
| **Skilled agricultural & fishery workers** | 402.5 | 385.5 | 373.5 | 374.5 | 299.6 | 300.4 | 294.4 | 292.7 | 290.6 | 298.6 | 293,2 | 270,0 | 262,0 | 264,8 |
| **Craft & related trade workers** | 571.8 | 587.4 | 594.8 | 609.8 | 598.6 | 605.2 | 594.5 | 631.2 | 606.6 | 568.1 | 505,4 | 438,9 | 366,2 | 320,1 |
| **Plant & machine operators & assemblers** | 283.4 | 278.4 | 281.0 | 285.4 | 293.2 | 293.1 | 310.7 | 302.9 | 302.3 | 305.9 | 284,8 | 231,6 | 210,7 | 199,2 |
| **Elementary occupations** | 114.8 | 122.0 | 126.0 | 138.5 | 122.2 | 124.1 | 135.3 | 131.9 | 141.8 | 149.5 | 154,2 | 126,8 | 108,8 | 98,7 |

**Table S2.** Female population (in thousands) by occupational group during the study period (2000–2013).

| **Category of profession** | **2000** | **2001** | **2002** | **2003** | **2004** | **2005** | **2006** | **2007** | **2008** | **2009** | **2010** | **2011** | **2012** | **2013** |
| --- | --- | --- | --- | --- | --- | --- | --- | --- | --- | --- | --- | --- | --- | --- |
| **Armed forces (unclassified persons)** | 2.8 | 2.6 | 4.1 | 3.2 | 5.1 | 5.5 | 6.5 | 5.9 | 5.1 | 7.5 | 8,81 | 5,9 | 5,5 | 9,5 |
| **Managers, executives, directors (members)** | 102.4 | 96.5 | 106.9 | 106.1 | 117.6 | 119.6 | 122.3 | 126.5 | 134.8 | 137.8 | 132,9 | 39,5 | 41,6 | 51,3 |
| **Professionals** | 225.6 | 236.5 | 248.9 | 258.9 | 285.5 | 290.2 | 305.9 | 314.1 | 329.7 | 332 | 341,9 | 367,4 | 349,2 | 349,5 |
| **Technologists & associate professionals** | 127.6 | 126.9 | 140.1 | 152.2 | 159.5 | 171 | 186.4 | 191.4 | 192.5 | 197.7 | 197,9 | 157,3 | 151,5 | 133,9 |
| **Clerks** | 264.8 | 272.9 | 260.6 | 266.4 | 291.9 | 288.9 | 307.4 | 305.3 | 313.2 | 290.5 | 283,7 | 239,5 | 212,5 | 196,1 |
| **Service workers & market sale workers** | 269.1 | 278.3 | 285.9 | 306.9 | 316.9 | 320.1 | 325.7 | 345.1 | 361.3 | 363.6 | 353,9 | 442,1 | 382,8 | 338,5 |
| **Skilled agricultural & fishery workers** | 289.5 | 261.8 | 264.6 | 267.5 | 221.6 | 223.8 | 220.1 | 209.2 | 203.2 | 206.9 | 213,7 | 203,6 | 191,1 | 188,4 |
| **Craft & related trade workers** | 87 | 81.9 | 75.3 | 74.8 | 58.9 | 63.5 | 63.1 | 58.8 | 47.9 | 47.5 | 44,9 | 40,7 | 33,7 | 30,9 |
| **Plant & machine operators & assemblers** | 27.9 | 26.5 | 28.9 | 29.7 | 32.9 | 32.2 | 28.8 | 28.1 | 28.5 | 26.5 | 25,9 | 20,4 | 17,6 | 19,7 |
| **Elementary occupations** | 118.3 | 128.2 | 145.6 | 145.5 | 152.1 | 157.1 | 158.6 | 164.9 | 167.5 | 180.7 | 185,3 | 167,7 | 141,2 | 130,2 |

**Table S3.** Total CMRs (and 95% CI) due to suicide by occupational group in males and females aged 15–59 years during the pre-crisis (2000–2009*) and the total study period (2000-2013).

| Category of profession | Males (2000-2009)* | Males (2000-2013) | Females (2000-2009)* | Females (2000-2013) |
| --- | --- | --- | --- | --- |
| Armed forces (unclassified persons) | **2.02 (1.50–2.68)** | **1.56 (1.20–1.99)** | — | 0.96 (0.05-4.73) |
| Managers, executives, directors | 0.85 (0.72–1.00) | 0.96 (0.84–1.09) | 1.24 (0.74–1.96) | 1.29 (0.84–1.91) |
| Professionals | 0.50 (0.40–0.62) | 0.49 (0.41–0.57) | 1.07 (0.76–1.47) | 1.04 (0.79–1.32) |
| Technologists & associate professionals | 0.52 (0.39–0.69) | 0.45 (0.35–0.57) | 0.54 (0.27–0.97) | 0.63 (0.39–0.97) |
| Clerks | **2.13 (1.87–2.41)** | **2.23 (2.01–2.46)** | 1.27 (0.93–1.70) | **1.33 (1.03–1.68)** |
| Service workers & market sale workers | 0.72 (0.59–0.87) | 0.58 (0.49–0.67) | 0.34 (0.18–0.58) | 0.26 (0.15–0.41) |
| Skilled agricultural & fishery workers | **1.86 (1.65–2.08)** | **1.77 (1.61–1,95)** | 1.06 (0.70–1.55) | 1.09 (0.79–1.48) |
| Craft & related trade workers | 0.64 (0.55–0.73) | 0.65 (0.58–0.73) | 0.48 (0.13–1.21) | 0.48 (0.17–1.06) |
| Plant & machine operators & assemblers | 0.57 (0.46–0.69) | 0.53 (0.44–0.63) | 0.27 (0.01–1.05) | 0.60 (0.15–1.63) |
| Elementary occupations | **2.39 (2.05–2.78)** | **2.08 (1.82–2.37)** | 0.56 (0.28–1.00) | 0.49 (0.28–0.80) |

CMR, comparative mortality ratio; CI, confidence interval; in light gray occupational groups with increasing trend in CMR during the crisis period.

*Data published in Alexopoulos et al., 2016 (reference 13).

**Table S4a.** Comparative mortality ratios (and 95% confidence intervals) due to suicide by occupational group in males during 2000–2009.

| Occupation | Age groups | 2000 | 2001 | 2002 | 2003 | 2004 | 2005 | 2006 |
| --- | --- | --- | --- | --- | --- | --- | --- | --- |
| **Armed forces (unclassified persons)** | 15–39 | 2.65 (0.84–6.38) | 3.79 (1.39–8.41) | 5.68 (2.30–11.81) | 1.65 (0.28–5.45) | 2.29 (0.73–5.54) | 0.53 (0.03–2.63) | 1.65 (0.42–4.49) |
|  | 40–49 | * | * | 5.17 (0.87–17.07) | 1.55 (0.08–7.64) | 1.39 (0.07–6.83) | * | 1.20 (0.06–5.93) |
|  | 50–59 | * | 11.36 (0.57–56.04) | 25.97 (4.35–85.81) | * | * | 7.63 (0.38–37.65) | * |
| **Members** | 15–39 | 0.92 (0.37–1.92) | 0.38 (0.06–1.25) | 1.33 (0.54–2.78) | 1.30 (0.52–2.70) | 0.90 (0.29–2.06) | 1.03 (0.42–2.14) | 1.35 (0.62–2.56) |
|  | 40–49 | 0.19 (0.01–0.93) | 1.28 (0.56–2.54) | 0.60 (0.15–1.64) | 0.39 (0.10–1.05) | 0.58 (0.18–1.41) | 1.37 (0.64–2.60) | 0.77 (0.31–1.61) |
|  | 50–59 | 0.63 (0.16–1.71) | 1.21 (0.49–2.51) | 1.25 (0.46–2.78) | 0.81 (0.30–1.79) | 0.43 (0.07–1.44) | 0.76 (0.24–1.83) | 0.79 (0.29–1.74) |
| **Professionals** | 15–39 | 0.53 (0.14–1.45) | 0.61 (0.16–1.73) | 0.94 (0.26–2.28) | 0.84 (0.26–2.03) | 0.37 (0.06–1.24) | 0.17 (0.01–0.84) | 0.30 (0.05–1.00) |
|  | 40–49 | 0.40 (0.07–1.32) | 0.51 (0.13–1.39) | 0.66 (0.17–1.79) | 0.69 (0.25–1.53) | 0.44 (0.11–1.21) | 0.68 (0.22–1.64) | 0.50 (0.16–1.21) |
|  | 50–59 | 1.56 (0.63–3.24) | 0.93 (0.30–2.25) | 0.31 (0.02–1.52) | 0.18 (0.01–0.88) | 0.46 (0.08–1.51) | 0.59 (0.15–1.62) | 0.36 (0.06–1.18) |
| **Technologists & associate professionals** | 15–39 | 0.43 (0.07–1.43) | 0.52 (0.09–1.70) | 0.28 (0.01–1.39) | 0.71 (0.18–1.93) | 0.25 (0.01–1.24) | * | 0.59 (0.15–1.62) |
|  | 40–49 | 0.89 (0.15–2.96) | * | 0.45 (0.02–2.22) | 0.82 (0.21–2.23) | * | 1.77 (0.65–3.92) | * |
|  | 50–59 | 0.76 (0.04–3.73) | 1.45 (0.24–4.78) | 0.88 (0.04–4.33) | 1.09 (0.18–3.60) | 1.23 (0.21–4.08) | 1.62 (0.41–4.40) | 0.44 (0.02–2.17) |
| **Clerks** | 15–39 | 2.86 (1.70–4.55) | 1.42 (0.62–2.82) | 1.61 (0.65–3.36) | 1,63 (0.71–3,24) | 2.30 (1.17–4.09) | 1.66 (0.77–3.16) | 1.44 (0.63–2.84) |
|  | 40–49 | 2.06 (0.90–4.08) | 1.38 (0.51–3.06) | 3.27 (1.66–5.83) | 2.05 (1.04–3.66) | 1.72 (0.75–3.40) | 1.92 (0.84–3.79) | 1.81 (0.88–3.31) |
|  | 50–59 | 3.34 (1.55–6.34) | 2.67 (1.17–5.29) | 5.97 (3.03–10.64) | 2.81 (1.31–5.34) | 2.98 (1.30–5.90) | 0.68 (0.11–2.24) | 2.61 (1.21–4.96) |
| **Service workers. market sale workers** | 15–39 | 0.40 (0.13–1.00) | 0.57 (0.21–1.25) | 0.58 (0.18–1.40) | 0.93 (0.43–1.77) | 1.27 (0.64–2.26) | 0.21 (0.04–0.70) | 0.77 (0.33–1.51) |
|  | 40–49 | 1.04 (0.26–2.83) | * | 0.35 (0.01–1.73) | * | 1.04 (0.33–2.51) | 0.64 (0.11–2.13) | 0.67 (0.17–1.81) |
|  | 50–59 | * | 1.65 (0.42–4.50) | 0.67 (0.03–3.29) | 0.39 (0.02–1.92) | * | 2.20 (0.70–5.30) | 1.05 (0.18–3.47) |
| **Skilled agricultural & fishery workers** | 15–39 | 2.75 (1.77–4.10) | 1.59 (0.83–2.76) | 1.49 (0.69–2.83) | 2.80 (1.69–4.39) | 1.05 (0.38–2.33) | 2.64 (1.50–4.33) | 2.88 (1.67–4.64) |
|  | 40–49 | 2.70 (1.50–4.51) | 0.84 (0.27–2.02) | 0.79 (0.20–2.16) | 2.21 (1.26–3.63) | 1.67 (0.77–3.17) | 0.49 (0.08–1.63) | 1.40 (0.61–2.76) |
|  | 50–59 | 1.52 (0.74–2.79) | 1.42 (0.69–2.61) | 0.68 (0.17–1.86) | 3.07 (1.97–4.57) | 2.72 (1.38–4.85) | 1.18 (0.43–2.62) | 2.21 (1.16–3.94) |
| **Craft & related trade workers** | 15–39 | 0.30 (0.11–0.66) | 1.31 (0.81–2.00) | 0.61 (0.27–1.21) | 0.07 (0.00–0.36) | 0.85 (0.45–1.48) | 0.62 (0.30–1.14) | 0.61 (0.30–1.12) |
|  | 40–49 | 0.80 (0.35–1.58) | 0.71 (0.31–1.41) | 0.11 (0.01–0.56) | 0.44 (0.18–0.92) | 0.92 (0.47–1.64) | 0.43 (0.13–1.03) | 1.22 (0.71–1.96) |
|  | 50–59 | 0.84 (0.34–1.74) | * | 0.66 (0.21–1.60) | 0.09 (0.00–0.46) | 0.55 (0.17–1.33) | 1.19 (0.60–2.12) | 1.12 (0.57–2.90) |
| **Plant & machine operators & assemblers** | 15–39 | 0.77 (0.31–1.60) | 1.05 (0.46–2.08) | 0.56 (0.14–1.53) | 0.16 (0.01–0.78) | 0.66 (0.21–1.58) | 0.30 (0.05–0,99) | 0.14 (0.01–0.68) |
|  | 40–49 | 0.22 (0.01–1.10) | 1.85 (0.90–3.40) | 0.77 (0.20–2.11) | 0.48 (0.12–1.30) | 0.34 (0.06–1.14) | 0.41 (0.07–1.35) | 0.44 (0.11–1.21) |
|  | 50–59 | 0.53 (0.09–1.77) | 1.24 (0.45–2.75) | 0.32 (0.01–1.57) | 0.41 (0.07–1.36) | 1.21 (0.38–2.92) | 0.73 (0.19–1.99) | 0.63 (0.16–1.71) |
| **Elementary occupations** | 15–39 | 3.12 (1.58–5.55) | 4.29 (2.39–7.15) | 2.75 (1.20–5.43) | 2.68 (1.31–4.91) | 2.14 (0.87–4.45) | 4.76 (2.77–7.68) | 2.10 (0.92–4.15) |
|  | 40–49 | 3.64 (1.48–7.58) | 3.39 (1.37–7.05) | 3.15 (1.16–6.99) | 2.24 (0.91–4.66) | 3.02 (1.22–6.28) | 1.14 (0.19–3.78) | 1.44 (0.46–3.47) |
|  | 50–59 | 0.64 (0.03–3.15) | 0.55 (0.03–2.70) | 0.79 (0.03–3.91) | 0.47 (0.02–2.31) | 3.04 (0.96–7.33) | 1.85 (0.47–5.04) | 1.48 (0.38–4.02) |

Data are presented as CMR (95% CI); CI, confidence interval; CMR, comparative mortality ratio. * No deaths by suicide registered to the Hellenic Statistical Authority.

**Table S4b.** Comparative mortality ratios (and 95% confidence intervals) due to suicide by occupational group in males during 2000–2009.

| Occupation | Age groups | 2007 | 2008 | 2009 | 2010 | 2011 | 2012 | 2013 |
| --- | --- | --- | --- | --- | --- | --- | --- | --- |
| **Armed forces (unclassified persons)** | 15–39 | 1.21 (0.20–4.02) | 3.24 (1.31–6.73) | 1.03 (0.17–3.41) | * | 1.63 (0.52-3.93) | 0.48 (0.02-2.39) | 1.30 (0.33-3.52) |
|  | 40–49 | 1.24 (0.06–6.11) | * | 1.27 (0.06–6.25) | 1.02 (0.05-5.04) | * | 0.69 (0.03-3.41) | 0.78 (0.04-3.84 |
|  | 50–59 | 10.75 (1.80–35.52) | * | 5.18 (0.26–25.55) | 2.65 (0.13-13.08) | * | * | * |
| **Members** | 15–39 | 0.22 (0.01–1.07) | 0.75 (0.24–1.80) | 1.33 (0.58–2.62) | 1.31 (0.53-2.73) | 1.38 (0.35-3.75) | 2.77 (1.02-6.14) | 0.89 (0.15-2.93) |
|  | 40–49 | 0.79 (0.32–1.64) | 0.52 (0.16–1.26) | 0.81 (0.33–1.69) | 0.53 (0.17-1.29) | 1.65 (0.72 -3.27 | 2.35 (1.31-3.92) | 1.30 (0.52-2.70) |
|  | 50–59 | 0.92 (0.34–2.05) | 0.64 (0.20–1.55) | 1.40 (0.74–2.43) | 0.84 (0.37-1.66) | 1.83 (0.85-3.47) | 2.16 (1.00-4.10) | 0.40 (0.07-1.34) |
| **Professionals** | 15–39 | 0.19 (0.01–0.92) | 0.34 (0.06–1.11) | 0.63 (0.19–1.51) | 0.53 (0.13-1.44) | 0.28 (0.05-0.91) | 0.57 (0.18-1.38) | 0.28 (0.05-0.91) |
|  | 40–49 | * | 0.67 (0.24–1.48) | 0.55 (0.18–1.34) | 0.14 (0.01-0.67) | 0.20 (0.03-0.67) | 0.57 (0.25-1.12) | 0.28 (0.07-0.75) |
|  | 50–59 | 0.39 (0.06–1.29) | 0.82 (0.30–1.82) | 0.13 (0.01–0.64) | 0.45 (0.14-1.07) | 0.67 (0.31-1.27) | 0.73 (0.32-1.44) | 0.69 (0.32-1.30) |
| **Technologists & associate professionals** | 15–39 | * | 0.21 (0.01–1.03) | 0.38 (0.06–1.26) | 0.82 (0.26-1.97) | 0.69 (0.17-1.87) | 0.27 (0.01-1.34) | 0.27 (0.01-1.36) |
|  | 40–49 | 0.45 (0.07–1.47) | 0.23 (0.01–1.13) | 0.80 (0.20–2.19) | 0.25 (0.01-1.23) | 0.22 (0.01-1.06) | 0.38 (0.06-1.25) | 0.22 (0.01 -1,07) |
|  | 50–59 | 1.53 (0.39–4.17) | 0.45 (0.02–2.23) | 0.69 (0.11–2.26) | * | 0.23 (0.01-1.12) | * | 0.25 (0.01-1.22) |
| **Clerks** | 15–39 | 2.40 (1.17–4.41) | 1.11 (0.41–2.46) | 1.99 (0.97–3.65) | 3.81 (2.26-6.06) | 1.64 (0.76-3.12) | 2.58 (1.36-4.48) | 2.08 (0.97-3.96) |
|  | 40–49 | 2.77 (1.54–4.62) | 2.32 (1.22–4.04) | 2.58 (1.36–4.48) | 2.43 (1.23-4.33) | 1.89 (0.92- 3.47) | 3.63 (2.28-5.51) | 2.12 (1.11-3.68) |
|  | 50–59 | 3.07 (1.42–5.82) | 0.63 (0.11–2.09) | 2.32 (1.13–4.27) | 2.04 (0.99-3.74) | 2.33 (1,29-3.88) | 2.31 (1.13-4.24) | 2.21 (1.08-4.05) |
| **Service workers. market sale workers** | 15–39 | 0.76 (0.31–1.58) | 0.66 (0.27–1.38) | 0.41 (0.13–1.00) | 0.34 (0.08-0.91) | 0.55 (0.27-1.09) | 0.42 (0.15-0.94) | 0.57 (0.25-1.14) |
|  | 40–49 | 1.57 (0.69–3.11) | 1.27 (0.51–2.64) | 0.61 (0.15–1.65) | 1.25 (0.50-2.59) | * | 0.07 (0.00-0.36) | 0.53 (0.21-1.09) |
|  | 50–59 | 0.55 (0.03–2.73) | 3.59 (1.57–7.10) | 0.69 (0.12–2.29) | 1.26 (0.40-3.03) | 0.22 (0.04-0.71) | 0.14 (0.01-0.67) | 0.25 (0.04-0.82) |
| **Skilled agricultural & fishery workers** | 15–39 | 2.20 (1.07–4.03) | 1.48 (0.65–2.92) | 2.51 (1.36–4.26) | 1.69 (0.74-3.33) | 1.03 (0.38-2.29) | 1.50 (0.65-2.96) | 1.46 (0.64-2.88) |
|  | 40–49 | 2.16 (1.13–3.75) | 1.00 (0.37–2.21) | 2.05 (1.08–3.56) | 1.78 (0.90-3.17) | 2.14 (1.19-3.56) | 1.12 (0.54-2.05) | 1.17 (0.57-2.15) |
|  | 50–59 | 1.17 (0.43–2.59) | 1.50 (0.65–2.97) | 1.80 (0.97–3.05) | 1.71 (0.95-2.85) | 1.91 (1.15-2.99) | 1.73 (0.96-2.88) | 1.83 (1.10-2.87) |
| **Craft & related trade workers** | 15–39 | 0,52 (0,23-1,03) | 0.60 (0.29–1.11) | 0.89 (0.50–1.48) | 0.77 (0.38-1.42) | 0.32 (0.10-0.78) | 0.33 (0.08-0.91) | 0.72 (0.29-1.49) |
|  | 40–49 | 0.73 (0.36–1.35) | 0.54 (0.22–1.11) | 0.38 (0.12–0.92) | 0.88 (0.43-1.61) | 0.86 (0.42-1.57) | 0.54 (0.24-1.07) | 0.66 (0.29-1.31) |
|  | 50–59 | 0.88 (0.39–1.74) | 0.36 (0.09–0.99) | 0.74 (0.34–1.41) | 0.65 (0.28-1.28) | 0.78 (0.39-1.39) | 0.95 (0.46-1.75) | 0.74 (0.32-1.46) |
| **Plant & machine operators & assemblers** | 15–39 | 0.68 (0.22–1.65) | 0.75 (0.28–1.67) | 0.14 (0.01–0.69) | 0.33 (0.06-1.10) | 0.18 (0.01-0.88) | 0.43 (0.07-1.42) | * |
|  | 40–49 | 0.63 (0.20–1.51) | 0.31 (0.05–1.03) | 0.47 (0.12–1.29) | 1.08 (0.47-2.14) | 0.44 (0.11-1.19) | 0.11 (0.01-0.56) | 0.25 (0.04--.81) |
|  | 50–59 | 0.25 (0.01–1.24) | 0.73 (0.18–1.98) | 0.50 (0.13–1.36) | 1.11 (0.48-2.20) | 0.28 (0.05-0.94) | 0.56 (0.14-1.53) | 0.67 (0.21-1.63) |
| **Elementary occupations** | 15–39 | 2.89 (1.34–5.49) | 3.81 (2.17–6.25) | 1.90 (0.88–3.60) | 1.74 (0.76-3.44) | 1.20 (0.44-2.66) | 1.44 (0.53-3.20) | 0.97 (0.25-2.64) |
|  | 40–49 | 0.82 (0.14–2.69) | 2.73 (1.19–5.39) | 1.46 (0.46–3.51) | 1.03 (0.26-2.80) | 1.67 (0.61-3.70) | 1.73 (0.70-3.6) | 1.35 (0.43-3.26) |
|  | 50–59 | 1.77 (0.45–4.82) | 2.31 (0.73–5.78) | 2.20 (0.81–4.88) | 1.82 (0.67-4.04) | 0.74 (0.12-2.45) | 2.15 (0.68-5.19) | 2.15 (0.79-4.77) |

Data are presented as CMR (95% CI); CI, confidence interval; CMR, comparative mortality ratio. * No deaths by suicide registered to the Hellenic Statistical Authority.

**Table S5a.** Comparative mortality ratios (and 95% confidence intervals) due to suicide by occupational group in females during 2000–2009.

| **Occupation** | **Age groups** | **2000** | **2001** | **2002** | **2003** | **2004** | **2005** | **2006** |
| --- | --- | --- | --- | --- | --- | --- | --- | --- |
| **Members** | 15–39 | 1.56 (0.09–7.72) | 7.94 (1.33–26.22) | * | * | 8.26 (2.62–19.93) | 1.40 (0.07–6.89) | * |
|  | 40–49 | * | 3.26 (0.16–16.06) | * | 3.55 (0.60–11.74) | * | * | * |
|  | 50–59 | * | 3.03 (0.15–14.95) | * | * | * | * | * |
| **Professionals** | 15–39 | 0.49 (0.02–2.40) | 1.15 (0.06–5.67) | 1.55 (0.26–5.13) | 1.59 (0.41–4.34) | 1.67 (0.34–4.72) | 0.40 (0.02–1.97) | 1.18 (0.20–3.91) |
|  | 40–49 | 1.76 (0.09–8.67) | 1.41 (0.07–6.95) | 1.69 (0.28–5.58) | 0.90 (0.04–4.43) | 1.68 (0.28–5.57) | 0.54 (0.03–2.65) | * |
|  | 50–59 | * | 2.39 (0.12–11.77) | * | 2.00 (0.10–9.89) | 2.26 (0.11–11.13) | 5.26 (0.88–17.39) | 4.26 (1.35–10.27) |
| **Technologists & associate-professionals** | 15–39 | 2.17 (0.55–5.90) | * | 1.15 (0.05–5.65) | * | * | 0.57 (0.03–2.82) | 0.85 (0.04–4.19) |
|  | 40–49 | * | * | 3.86 (0.65–12.76) | * | * | 1.07 (0.05–5.28) | * |
|  | 50–59 | * | * | * | * | * | * | * |
| **Clerks** | 15–39 | 0.38 (0.02–1.88) | 2.74 (0.70–7.45) | 1.33 (0.22–4.38) | 1.33 (0.33–3.62) | 0.99 (0.16–3.26) | 1.09 (0.28–2.97) | 0.52 (0.03–2.58) |
|  | 40–49 | 1.62 (0.08–8.01) | * | 0.91 (0.04–4.50) | * | * | 1.70 (0.43–4.64) | * |
|  | 50–59 | 4.56 (0.76–15.05) | 2.82 (0.14–13.93) | 6.37 (0.32–31.41) | 10.18 (2.23–24.55) | 3.02 (0.15–14.9) | 3.53 (0.18–17.43) | 1.52 (0.07–7.48) |
| **Service workers, market sale workers** | 15–39 | 0.37 (0.02–1.85) | * | 0.61 (0.03–3.02) | * | * | 0.62 (0.10–2.06) | 0.48 (0.02–2.39) |
|  | 40–49 | 1.92 (0.09–9.47) | * | 0.96 (0.05–4.72) | 1.05 (0.05–5.18) | * | * | * |
|  | 50–59 | * | * | * | * | * | * | * |
| **Skilled agricultural & fishery workers** | 15–39 | * | * | * | 2.25 (0.38–7.42) | 1.67 (0.08–8.25) | 1.25 (0.06–6.16) | 3.52 (0.59–11.63) |
|  | 40–49 | * | * | * | * | 2.37 (0.40–7.82) | 1.60 (0.27–5.28) | * |
|  | 50–59 | 0.51 (0.02–2.52) | 0.79 (0.04–3.89) | 1.81 (0.09–8.93) | 1.57 (0.26–5.20) | * | 1.49 (0.07–7.37) | 0.67 (0.03–3.08) |
| **Craft & related trade workers** | 15–39 | * | * | * | * | 3.73 (0.19–18.4) | * | * |
|  | 40–49 | * | * | * | * | * | * | * |
|  | 50–59 | * | * | * | * | * | * | * |
| **Plant & machine operators & assemblers** | 15–39 | * | * | * | * | * | * | * |
|  | 40–49 | * | * | * | * | * | * | * |
|  | 50–59 | * | * | * | * | * | * | * |
| **Elementary occupations** | 15–39 | * | * | * | 1.30 (0.06–6.43) | 1.50 (0.07–7.40) | * | 1.83 (0.09–9.03) |
|  | 40–49 | * | * | * | 1.36 (0.07–6.72) | 1.20 (0.06–5.94) | 0.82 (0.04–4.05) | * |
|  | 50–59 | * | * | * | * | * | * | * |

Data are presented as CMR (95% CI); CI, confidence interval; CMR, comparative mortality ratio. * No deaths by suicide registered to the Hellenic Statistical Authority.

**Table S5b.** Comparative mortality ratios (and 95% confidence intervals) due to suicide by occupational group in females during 2000–2009.

| **Occupation** | **Age groups** | **2007** | **2008** | **2009** | **2010** | **2011** | **2012** | **2013** |
| --- | --- | --- | --- | --- | --- | --- | --- | --- |
| **Members** | 15–39 | 1.97 (0.10–9.71) | * | 2.06 (0.08–10.41) | 3.04 (0.15-14.99) | * | * | * |
|  | 40–49 | * | 0.91 (0.04–4.49) | 1.67 (0.08–8.23) | * | 4.53 (0.23-22.32) | * | 2.20 (0.11-10.86) |
|  | 50–59 | * | 3.99 (0.67–13.19) | * | * | 8.62 (0.43-42.52) | 4.54 (0.23-22.42) | * |
| **Professionals** | 15–39 | 1.52 (0.38–4.12) | * | 1.15 (0.19–3.79) | * | 1.07 (0.34-2.57) | 1.40 (0.45-3.38) | 0.66 (0.11-2.17) |
|  | 40–49 | 0.97 (0.05–4.80) | 0.39 (0.02–1.97) | * | 1.05 (0.05-5.17) | * | 1.09 (0.18-3.62) | 1.15 (0.29-3.12) |
|  | 50–59 | * | 1.29 (0.06–6.38) | * | 5.41 (0.91-17.86) | 1.31 (0.06-6.46) | * | 1.75 (0.44-4.75) |
| **Technologists & associate-professionals** | 15–39 | * | * | * | 1.07 (0.05-5.27) | 0.52 (0.03-2.58) | 0.67 (0.03-3.30) | * |
|  | 40–49 | * | * | 1.47 (0.07–7.25) | * | 1.29 (0.06-6.35) | * | 0.97 (0.05-4.76) |
|  | 50–59 | 5.62 (0.28–27.71) | * | * | * | 8.66 (1.45-28.6) | 2.60 (0.13-12.84) | * |
| **Clerks** | 15–39 | 1.89 (0.60–4.55) | 2.96 (0.94–7.13) | 1.17 (0.19–3.86) | 1.66 (0.28-5.47) | 1.91 (0.70-4.23) | 1.49 (0.38-4.07) | 0.54 (0.03-2.68) |
|  | 40–49 | 1.04 (0.04–5.15) | 0.92 (0.15–3.05) | * | 2.81 (0.47-9.28) | 2.43 (0.62-6.62) | 2.93 (0.74-7.97) | * |
|  | 50–59 | * | * | 2.02 (0.10–9.94) | * | * | 3.23 (0.54-10.66) | * |
| **Service workers, market sale workers** | 15–39 | 0.78 (0.13–2.58) | * | 0.44 (0.02–2.15) | * | 0.20 (0.01-1.01) | * | 0.30 (0.01-1.50) |
|  | 40–49 | * | * | * | * | * | * | * |
|  | 50–59 | * | 1.53 (0.07–7.54) | 1.80 (0.07–9.08) | * | * | * | 0.71 (0.03-3.5) |
| **Skilled agricultural & fishery workers** | 15–39 | 1.68 (0.08–8.27) | * | 2.34 (0.11–11.55) | * | 1.11 (0.06-5.45) | * | * |
|  | 40–49 | 1.68 (0.08–8.29) | 2.31 (0.59–6.28) | 1.33 (0.07–6.55) | 2.00 (0.10-9.84) | * | 1.21 (0.06-5.98) | * |
|  | 50–59 | * | 3.19 (0.81–8.69) | * | * | 4.77 (1.51-11.5) | 0.81 (0.04-3.98) | 2.14 (0.68-5.15) |
| **Craft & related trade workers** | 15–39 | * | * | * | * | * | * | * |
|  | 40–49 | 4.24 (0.21–20.90) | * | 5.24 (0.26–25.82) | * | * | * | * |
|  | 50–59 | * | 6.49 (0.32–32.03) | * | * | * | 6.67 (0.33-32.88) | * |
| **Plant & machine operators & assemblers** | 15–39 | * | 10.99 (0.55–54.20) | * | 12.66 (0.63-62.43) | * | * | * |
|  | 40–49 | * | * | * | * | * | * | * |
|  | 50–59 | * | * | * | 28.57 (1.43-140.9) | * | * | * |
| **Elementary occupations** | 15–39 | * | * | 2.97 (0.50–9.80) | * | * | * | 1.19 (0.06-5.88) |
|  | 40–49 | * | * | * | * | * | * | * |
|  | 50–59 | * | * | 6.05 (1.54–16.46) | * | 1.73 (0.09-8.53) | 1.40 (0.07-6.89) | * |

Data are presented as CMR (95% CI); CI, confidence interval; CMR, comparative mortality ratio. * No deaths by suicide registered to the Hellenic Statistical Authority.
